# A SWOT Analysis of the Use and Potential Misuse of Implantable Monitoring Devices by Athletes

**DOI:** 10.3389/fphys.2017.00629

**Published:** 2017-09-05

**Authors:** Billy Sperlich, Peter Düking, Hans-Christer Holmberg

**Affiliations:** ^1^Integrative and Experimental Exercise Science, Institute for Sport Sciences, University of Würzburg Würzburg, Germany; ^2^Swedish Winter Sports Research Centre, Mid Sweden University Östersund, Sweden; ^3^School of Sport Sciences, UiT The Arctic University of Norway Tromsø, Norway; ^4^School of Kinesiology, University of British Columbia Vancouver, BC, Canada

**Keywords:** implant, implantable neurostimulators, ingestible sensor, sensor assessment, athletes

We have been following the developments and popularity of commercially available wearable sensor technology, as well as the ongoing discussion concerning its usefulness for improving the fitness and health of athletes (Düking et al., 2016, 2017; Sperlich and Holmberg, 2017) with considerable interest.

Here, we would like to draw attention to a new generation of implantable devices (implantables) currently being promoted as “the next wave of sensor-based smart devices” (Khosravi, 2015) and predicted to be “a big thing in three years” (Mercer, 2016). We perform a SWOT analysis regarding their use, especially by athletes and in sports, with the goal of identifying internal strengths and weaknesses, as well as external opportunities and threats.

The primary SWOT matrix employed is illustrated in Figure 1.

**Figure 1:**
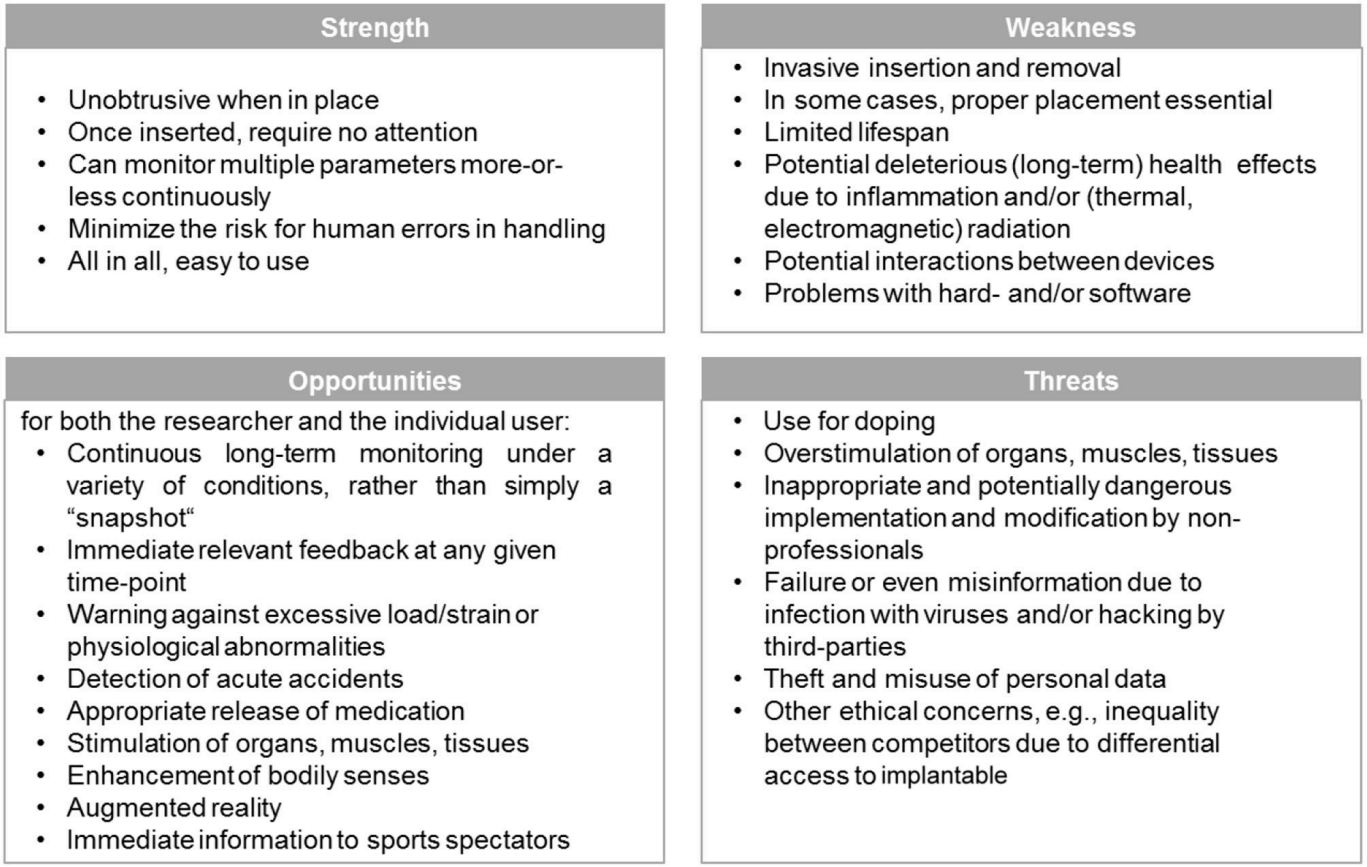
SWOT matrix for identifying the strengths and weaknesses, as well as opportunities and threats of implantables.

## Strengths

Once inserted, implantables are inherently unobtrusive and without being noticed, can monitor a plethora of internal and external parameters and transmit this information to other devices or use it to stimulate human tissue(s) with electrical, chemical, acoustical, visual and/or (vibro-)tactile signals. Once securely implanted, the devices can be forgotten and handling errors do not occur, thereby combining minimal loss of data with ease of use.

## Weaknesses

Implantable sensors must be inserted and, at some point, removed more-or-less invasively, requiring minor or even major surgery, depending on the device and its anatomical positioning. Invasive removal may be required due to restricted chip life (currently expected to be around 10 years; Catherwood and McLaughlin, 2015), limited battery charge, loosening or damage (e.g., in contact sports such as, rugby or boxing) or simply malfunction. Moreover, in most sports, where competitive margins are small, athletes want and need the most up-to-date hardware, and in accordance with Moore's Law chips are presently becoming more powerful with time (Waldrop, 2016). Thus, athletes will probably choose to replace fully functional hardware with the newest model.

## Opportunities

Implantable sensors allow continuous long-term monitoring of various parameters concerning an athlete's health and training, thereby providing more holistic information than performance and health diagnostics usually performed only once (Düking et al., 2016). Implantables can also detect acute accidents, such as, concussions sustained in connection with boxing and rugby, thereby helping to reduce the seriousness of injuries. In addition, they will be able to release substances automatically and in proper doses when needed, enhancing performance and aiding recovery, rendering certain types of delayed medication obsolete.

Furthermore, electrodes implanted within the muscle may stimulate adaptation in a beneficial manner.

Vision and hearing can already be enhanced with contact lenses and hearing aids, respectively, and further elevation of bodily senses is to be expected. Moreover, with augmented reality, tactical options such as, the optimal pass in team sports can actually be displayed on the retina of the athlete, speeding up and optimizing play.

In addition to opportunities related directly to the athlete, implantables in sports provide opportunities for manufacturers. As already carried out to perfection in Formula 1 racing, sensors which monitor aspects of a sport allow manufacturers to test and improve their products employing the best athletes. Simultaneously, broadcasting the parameters monitored to spectators enhances both their involvement and enjoyment of the game (Fuss, 2014).

## Threats

Obviously, implantables can potentially be used for doping, administering small, undetectable doses of a substance during periods when controls are not being performed. Since these devices are relatively new, procedures for detecting such doping have yet to be developed. Normally, a substance/device is detected only on the basis of its known identity and working principle, but it may be possible to detect certain implantable sensors on the basis of the thermic and/or electromagnetic radiation they emit.

Likewise, signals from implantables could lead to (unintentional or intentional) overstimulation of human tissues, and (thermic) radiation passing and/or dissipate through tissues/organs, may compromise the athlete's long-term health (Catherwood and McLaughlin, 2015).

As implantables become smaller, cheaper, and more commercially available, non-professionals may begin to modify and implant devices into their skin (like piercing) or other organs for inappropriate reasons, increasing the likelihood of deleterious health effects (Catherwood and McLaughlin, 2015).

The software in implantables can be hacked or infected by viruses by third-parties, e.g., competitors, threating the security of personal data and health and potentially providing an unfair advantage to the opponent. Even with advanced encryption technology, devices can still be hacked (Austen, 2015) and it is unlikely that they will ever be completely secure.

Further ethical questions in this context include: (i) Who decides whether an implantable is to be inserted (athlete, coach, team management, league management)? (ii) Who owns the information provided by an implantable? (iii) Should the information provided by implantables be used to decide which athletes to employ? (iv) What aspects are allowed to be monitored and when and does this compromise the athlete's integrity? (v) Who sets the magnitude and frequency of implantable stimulation of tissues?

To summarize, due to their ease of handling and numerous valuable applications, implantables can potentially improve health, help prevent disease and improve performance. At the same time, the involvement of powerful commercial interests, including the digital, pharmaceutical, bionic, medical, and sporting industries, makes critical evaluation and regulation of potential misuse imperative. The technology must be safe, employed only for therapeutic and preventive purposes, and installed and monitored only by professionals. There is a tremendous risk for clandestine misuse of ergogenic technology (e.g., for doping) by athletes, which is especially disturbing in light of our lack of knowledge of the long-term psychological and physiological effects. Protection of both the athlete and patient is paramount and we must formulate appropriate rules and ethical standards, especially concerning enhancement of athletic prowess.

## Author contributions

All authors listed have made substantial, direct and intellectual contribution to this work and approved it for publication.

### Conflict of interest statement

The authors declare that the research was conducted in the absence of any commercial or financial relationships that could be construed as a potential conflict of interest.
